# Comparative analysis of the integument transcriptomes of the *black dilute* mutant and the wild-type silkworm *Bombyx mori*

**DOI:** 10.1038/srep26114

**Published:** 2016-05-19

**Authors:** Songyuan Wu, Xiaoling Tong, Chenxing Peng, Gao Xiong, Kunpeng Lu, Hai hu, Duan Tan, Chunlin Li, Minjin Han, Cheng Lu, Fangyin Dai

**Affiliations:** 1 State Key Laboratory of Silkworm Genome Biology, Key Laboratory for Sericulture Functional Genomics and Biotechnology of Agricultural Ministry, Southwest University, Chongqing 400715, China

## Abstract

The insect cuticle is a critical protective shell that is composed predominantly of chitin and various cuticular proteins and pigments. Indeed, insects often change their surface pigment patterns in response to selective pressures, such as threats from predators, sexual selection and environmental changes. However, the molecular mechanisms underlying the construction of the epidermis and its pigmentation patterns are not fully understood. Among Lepidoptera, the silkworm is a favorable model for color pattern research. The *black dilute* (*bd*) mutant of silkworm is the result of a spontaneous mutation; the larval body color is notably melanized. We performed integument transcriptome sequencing of the wild-type strain Dazao and the mutant strains +/*bd* and *bd*/*bd*. In these experiments, during an early stage of the fourth molt, a stage at which approximately 51% of genes were expressed genome wide (RPKM ≥1) in each strain. A total of 254 novel transcripts were characterized using Cuffcompare and BLAST analyses. Comparison of the transcriptome data revealed 28 differentially expressed genes (DEGs) that may contribute to *bd* larval melanism, including 15 cuticular protein genes that were remarkably highly expressed in the *bd*/*bd* mutant. We suggest that these significantly up-regulated cuticular proteins may promote melanism in silkworm larvae.

Insects exhibit a high level of adaptability, an attribute that has assisted them in becoming the most numerous taxon on Earth. The insect epidermis provides an important basis for adaptation to the environment^1^. Indeed, the integument has evolved to both form a critical barrier that maintains the internal environment of the insect and to produce body coloring and markings^2^, with coloration evolving as a functional characteristic that helps insects avoid predators, carry out predatory behaviors, maintain immunity, and find mates^2^,^3^,^4^,^5^,^6^. Pigmentation often appears during insect development, with significant variation both intraspecifically and interspecifically^7^. In addition, alterations in color patterns follow changes in the surroundings. For example, in the industrial melanism of the peppered moth *Biston betularia* in Great Britain, the population of adults changed from wild-type (*typica*) to black (*carbonaria*)^8^. The buckeye butterfly *Junonia coenia* exhibits seasonal wing pigmentation plasticity: wing color is tan in spring and changes to dark red in autumn^9^. Similarly, young larvae of the swallowtail butterfly *Papilio xuthus* mimic bird droppings (mimetic pattern) and switch to a green camouflage coloration (cryptic pattern) during their final molting period^10^. Hence, coloration is considered one of the most variable characteristics among insects.

The silkworm *Bombyx mori* (Order Lepidoptera) emerged as a result of the domestication of the Chinese wild silkworm *B. mandarina* approximately 5000 years ago^11^. During the course of nearly 100 years of study on classical silkworm genetics, more than 600 mutant strains have been obtained and preserved in China and Japan^12^,^13^, including nearly 100 coloration mutant strains, providing a valuable resource for pigmentation research. Additionally, the silkworm is considered a potential model organism because of the recent completion of the sequencing of its genome^11^.

As a domesticated organism, the silkworm has undergone artificial selection with regard to color patterns, changing from a dark pigmentation to a light color. Physiological research has determined that the larval epidermis of *B. mori* contains a reduced amount of melanin; however, the number of urate granules, which accumulate in the dermis to maintain an opaque white epidermis, is increased^14^. The molecular mechanisms underlying changes in pigmentation patterns are a focus of genetics research. A previous study suggested that the *tyrosine hydroxylase* (*TH*) gene, which is upstream of the melanin synthesis pathway, may contribute to differences in body color between *B. mandarina* and *B. mori*^15^. The *black dilute* (*bd*) mutant, a spontaneous autosomal recessive mutation, was first reported in 1941 and was subsequently preserved in China and Japan. The *bd* larval body exhibits significantly more melanism than the wild-type Dazao and the heterozygote (+/*bd*) (Fig. 1), and the female moth is sterile. Classical genetic research on this mutant has identified it as a single-locus mutation linked to chromosome 9^16^. The *bd* mutant displays a dark larval body color similar to that of *B. mandarina* (Additional file 1: Fig. S1), and this prompted us to explore the molecular basis of body color in the *bd* mutant.

Previous research has indicated that coloration results from both regulatory and structural genes, with many of the structural genes encoding enzymes that are involved in the biochemical pathways that generate pigments^17^. *In vivo*, tyrosine undergoes a series of enzymatic reactions that generate the pigment precursors, DOPA, dopamine, N-acetyl dopamine (NADA) and N-β-alanyldopamine (NBAD). After further oxidation and translocation, DOPA and dopamine are converted into melanin and catecholamine derivatives and incorporated into the outer epidermis^18^,^19^. The key genes in this pathway include *tyrosine hydroxylase* (*TH*), *DOPA decarboxylase* (*DDC*), *N-acetyltransferase* (*AANAT*), *NBAD synthase* (*ebony*), NBAD hydrolase (*tan*)*, dopachrome-conversion enzyme* (*yellow-f*, *yellow-f2*), and melanism-promoting genes, *yellow, laccase 2*^20^,^21^(Additional file 2: Fig. S2). However, the details of this pathway are not completely understood, and they therefore require further research. Insects must molt because of the limited ductility of the exoskeleton, which is secreted by epidermal cells, and the coloration pattern is re-established following formation of a new epidermis during molting. Molting and metamorphosis are strictly regulated by ecdysone and juvenile hormones in metamorphosing insects^22^: high titers of ecdysone initiate the molting process, and genes that contribute to molting and color patterns are subsequently induced^23^,^24^. Thus, studying the changes that occur in gene expression during molting is an important approach to understanding the molecular mechanisms underlying molting and coloration. In silkworms, the fourth molting is a representative stage^25^. In the middle stage of molting (following head capsule slippage, HCS^25^), most of the structural genes, such as *yellow* and *ebony*^21^, in the pigment synthesis pathway are activated, whereas important regulatory genes are more likely to be activated during an earlier stage of molting. In this study, we aimed to detect early-expressed genes that guide molting, such as hormone-responsive genes and regulatory genes. In addition, we identified the genes that contribute to the *bd*/*bd* color pattern by comparing the transcriptomes of wild-type Dazao and *bd* mutant specimens.

## Results

### RNA sequencing and gene annotation

In metamorphosing insects, high titers of ecdysone will cause molting or metamorphosis^26^, and the characteristics exhibited during feeding activity and HCS are important indications of silkworm molting^25^. During this time, the expression levels of many genes are altered and increase in the ecdysone titer. We further aimed to investigate the expression of genes in the epidermis during an early stage of molting. We selected the wild-type Dazao strain and the mutants strains +/*bd* and *bd*/*bd*. The integument was collected from four individuals of each type of silkworm during the time that feeding was stopped, which was several hours before HCS, and the samples for each silkworm strain were then pooled. RNA from the epidermis of each pool was sequenced using an Illumina HiSeq 2000 platform. After performing quality control measures, 9.1 Gb, 9.2 Gb and 10.66 Gb of clean data were obtained for the Dazao, *bd*, and +/*bd* transcriptomes, respectively (Table 1). We mapped these clean reads (clean data) to the *B. mori* reference genome, version_2.0^27^, and the proportions of total reads that mapped to the genome were 91.75%, 81.24%, and 79.20% (Additional file 3: Table S1), respectively. All of the mapped reads obtained from the three silkworm types were merged and assembled using Cufflinks^28^. The genes were corrected based on known silkworm gene models that were identified in SilkDB (http://www.silkdb.org/silkdb/). These data were compared with SilkDB data, and a total of 2,157 potential novel transcripts were characterized using Cuffcompare. The locations of exons and introns in each novel transcript were also defined (Additional file 4: Table S2). These 2,157 potential new transcripts were compared using BLASTN against the silkworm transcriptome database (SilkTransDB)^29^, which includes transcriptomes obtained from specific tissues and from the whole body at various developmental stages, including embryos, larvae, pupae, and adult moths. The results showed a total of 1,903 similar transcripts in SilkTransDB, whereas for 254 of the novel transcripts, no corresponding transcripts were retrieved in a BLAST search using SilkTransDB (Additional file 4: Table S2). All of the new transcripts were analyzed using BLASTX against the non-redundant (nr) protein database (http://www.ncbi.nlm.nih.gov/), and all of genes and transcriptome data were analyzed using BLAST against the nr database to obtain functional annotations (Additional file 5: Table S3). We also analyzed the Gene Ontology (GO) classifications of 254 of the novel transcripts (Additional file 6: Fig. S3), and 80 were assigned to corresponding GO terms. We plotted the GO annotation results using the WEGO program (http://wego.genomics.org.cn/cgi-bin/wego/index.pl). The results included GO terms enriched in binding, cellular processes, metabolism processes, catalysis, cells and cell parts.

### Transcriptome profiles of the integument

The uniquely mapped reads were normalized and calculated using the reads per kilobase per million reads (RPKM) method to estimate the abundance of transcripts of each gene^30^. Genes with RPKMs of less than 1 were considered to be not expressed, and genes with RPKMs over 60 were considered to be expressed at very high levels. The distributions of the expression levels of all of the genes were similar across all three silkworm strains (Additional file 7: Fig. S4). Genome wide, approximately 51% of the genes were expressed (RPKM ≥1), with more than 6% highly expressed (RPKM >60), in each strain (Table 2).

**Figure 2:**
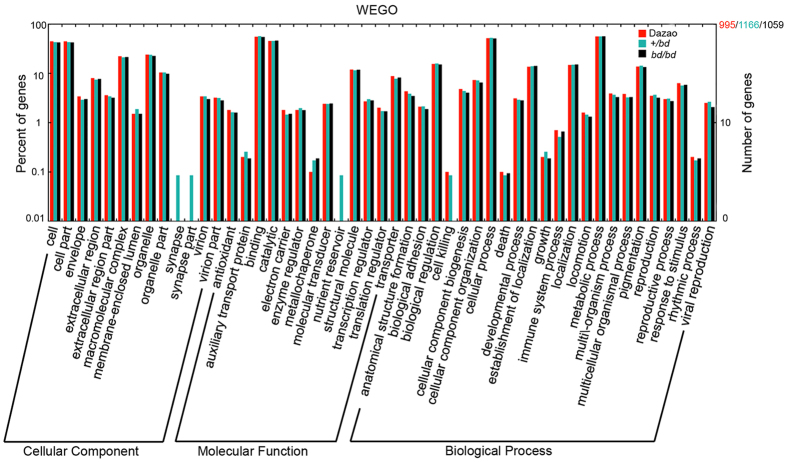
GO analysis of the highly expressed genes (RPKMs >60) that were identified in three strains. The x-axis shows the 2nd-level GO terms. The y-axis shows the percentage and number of genes: 995 genes in Dazao, 1166 genes in +/*bd*, and 1059 gens in *bd*/*bd.*

We analyzed the biological functions of the highly expressed genes (RPKM >60) using GO classifications. Based on results obtained using the Blast2GO program, 995, 1166, and 1059 highly expressed genes with GO terms were annotated for the Dazao, +/*bd* and *bd*/*bd* strains, respectively. The most highly enriched GO terms were cell, cell part, macromolecular complex, organelle, organelle part, binding, catalytic, structural molecular, biological regulation, cellular process, establishment of localization, localization, metabolic processes and pigmentation (Fig. 2). In addition, the GO terms synapse, synapse part, and nutrient reservoir were present only in the +/*bd* strain, and the term cell killing was absent in the *bd*/*bd* genotype. Among the genes that were linked to these GO terms, we identified *Novel00159* (predicted: pre-mRNA-processing-splicing factor 8) as being associated with the terms synapse and synapse part, and *BGIBMGA002611* (Predicted: lysine-specific demethylase 6A isoform X1) was identified as a nutrient reservoir, and the GO term cell killing was associated with *BGIBMGA011016* (predicted: organic cation transporter protein-like).

Moreover, *BGIBMGA013945* (*muscle actin*)*, BGIBMGA002259* (*myosin light chain*)*, BGIBMGA002715* (*nucleoporin*), and *BGIBMGA013700* (*troponin T*) were expressed at extremely high levels, and their RPKM values were >10,000 in all three genotypes.

### DEGs in the integument

**Figure 3:**
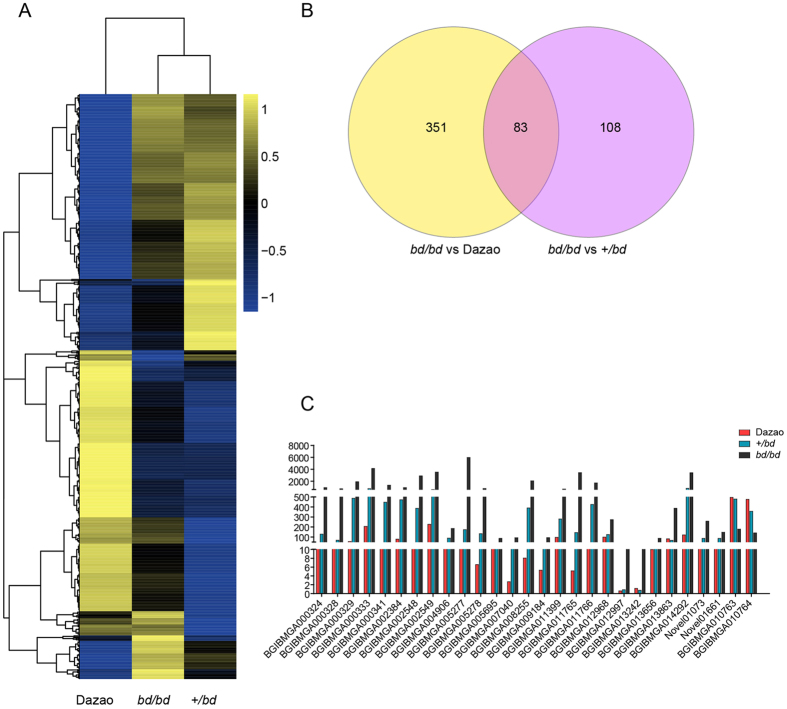
Analysis of DEGs. (**A**) A hierarchical clustering of DEGs was obtained using RNA-seq data that was derived from the three silkworm strains based on log_10_ RPKM values. The blue bands indicate low gene expression levels, and the yellow bands indicate high gene expression levels. (**B**) Venn diagram showing the overlaps between DEGs in the two sets (*bd*/*bd* vs. Dazao, *bd*/*bd* vs. +/*bd*). (**C**) Column chart of candidate DEGs. The red column indicates Dazao, the blue column indicates +/*bd*, and the black column indicates *bd*/*bd*. The Y-axis shows the transcription level.

To identify DEGs among the three silkworms using each sequenced library, the read counts were adjusted using the edgeR package with one normalized scaling factor^31^, and a differential expression analysis was performed using two samples with the DEGSeq R package^32^ (1.12.0). The P-values were adjusted using the Benjamin and Hochberg method, and a corrected P-value of 0.005 and a log_2_ (fold change) of 1 were set as the thresholds for significant differential expression. Dazao is considered to be a reference strain for silkworms, and its entire genome has been sequenced. In addition, *bd*/*bd* females are sterile, whereas +/*bd* females are fertile; thus, *bd* offspring are generated by crossing +/*bd* females with *bd*/*bd* males. Accordingly, after many years of crosses, *bd*/*bd* has become an inbred line of +/*bd* silkworms. We performed a comparative analysis of the transcriptome data derived from the *bd*/*bd* mutants and the two controls, Dazao and +/*bd*. All of the DEGs are shown in Additional file 8. To determine the expression patterns of the identified genes, we performed hierarchical clustering of all of the DEGs based on the log_10_ (RPKM+1) values for the three silkworms (Fig. 3A), generating a list of 191 DEGs between the *bd*/*bd* and +/*bd* strains, 434 DEGs between the *bd*/*bd* and Dazao strains, and 83 genes between these two sets of DEGs (Fig. 3B). We believe that the identified candidate DEGs, which potentially contribute to larval melanism, should exhibit similar patterns. Therefore, we excluded the genes that showed different patterns in the three silkworms. For example, the *BGIBMGA000023* gene, which encodes cecropin B, was expressed at a higher level (RPKMs = 393.62) in the *bd*/*bd* mutant than in the +/*bd* (RPKMs = 146.05) silkworm, but its expression level was lower than in the Dazao strain (RPKMs = 912.66). It is our opinion that the expression of candidate DEGs in the *bd*/*bd* strain should be either higher or lower than the expression in either of the controls (Dazao and +/*bd*). A total of 28 DEGs were sorted using manual screening (Fig. 3C). Of these, 26 DEGs were up-regulated in the *bd*/*bd* strain, and a BLAST search of the the nr and FlyBase^33^ databases indicated that 15 of these DEGs are cuticular proteins. The other 2 DEGs were down-regulated, and both were identified as aminopeptidase N-12 (Table 3). Based on our comparative analyses of transcriptome data and functional annotations, we suggest that these 28 DEGs may contribute to *bd*/*bd* color patterns.

### GO and KEGG pathway enrichment analysis of DEGs

**Figure 4:**
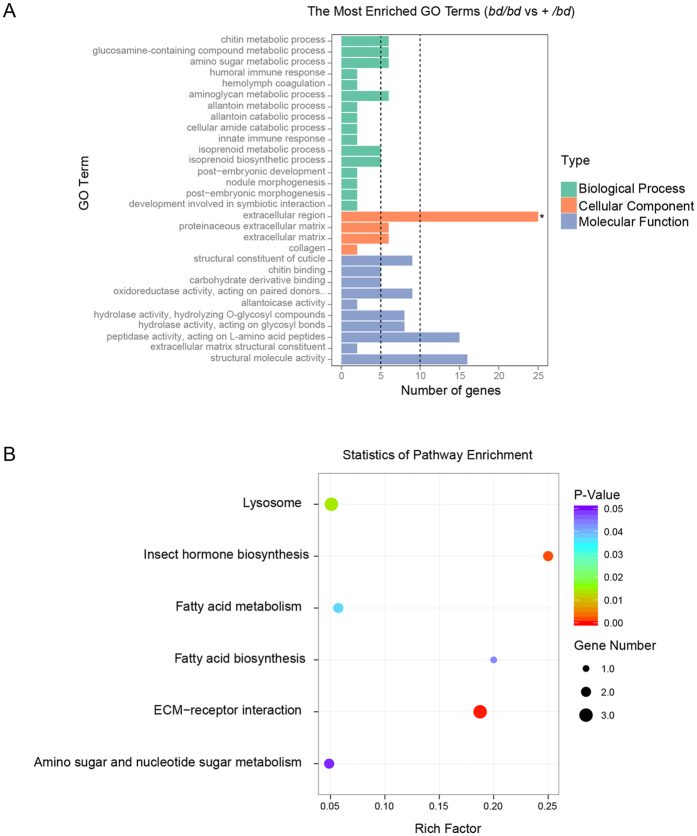
GO and KEGG enrichment analyses of DEGs. (**A**) GO enrichment analysis of DEGs (*bd*/*bd* vs. +/*bd*). The x-axis shows the number of genes, and the y-axis shows the GO terms. The green columns indicate the biological process category, the yellow columns indicate the cellular component category, and the purple columns indicate the molecular function category. (**B**) Scatterplot of enriched KEGG pathways for DEGs that were common between the *bd*/*bd* and +/*bd* strains. The enrichment factor indicates the ratio of the differentially expressed gene number to the total gene number in a certain pathway. The size and color of the dots represent the gene number and the range of P values, respectively.

To further analyze the identified DEGs, we performed GO and KEGG pathway analyses. Using a Blast2GO search, 10,118 genes or transcripts (43.45% of the total number of genes or transcripts) were annotated to identify GO terms, and a GO enrichment analysis of DEGs was implemented using the GOseq R package. In this analysis, gene length bias was corrected. GO terms with corrected P-values less than 0.05 were considered to be significantly enriched in the DEG analysis. Because *bd*/*bd* is an inbred line that is derived from +/*bd* silkworms, 191 of the DEGs were common between *bd*/*bd* and +/*bd*. A total of 123 of these 191 DEGs were annotated with GO terms, and the GO functional enrichment analysis of those genes revealed significantly enriched terms, including extracellular region, peptidase activity, structural molecule activity, structural constituent of cuticle, oxidoreductase activity, hydrolase activity, and chitin metabolic process (Fig. 4A). A KEGG pathway enrichment analysis was performed using KOBAS 2.0^34^, and the terms EMC-receptor, insect hormone biosynthesis, fatty acid biosynthesis, lysosome, fatty acid metabolism, amino sugar and nucleotide sugar metabolism (Fig. 4B) were significantly enriched (P-value < 0.05). Because there was a concern regarding the color of the epidermis, GO terms including structural constituent of cuticle, chitin metabolic process, and chitin binding, in addition to the KEGG term insect hormone biosynthesis, were selected. Using these criteria, 25 genes were detected (Additional file 9: Table S5), and these also included cuticular proteins, which were significantly highly expressed in *bd*/*bd* mutants.

### Validation of RNA-seq data using qRT-PCR

**Figure 5:**
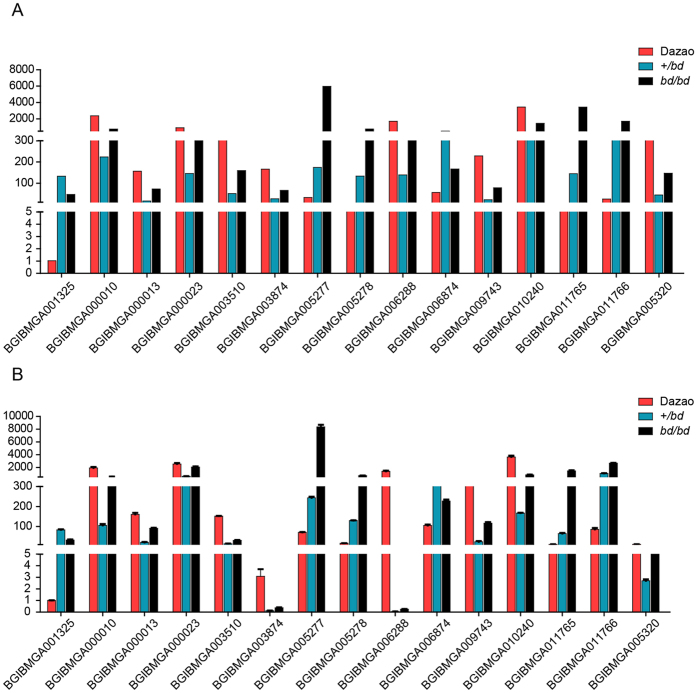
Relative gene expression. (**A**) Sequencing results. RPKM, reads per kb per million reads. The Y-axis indicates the transcription level. (**B**) Real-time quantitative PCR results. The Y-axis indicates the transcription level.

To validate the RNA-seq data, qRT-PCR was performed to analyze 15 randomly selected DEGs. We compared the RPKMs of the 15 DEGs, as shown in Fig. 5A. The expression levels were different between the three silkworms, and the results of qRT-PCR (Fig. 5B) were essentially consistent with the transcriptome data. For example, qRT-PCR for *BGIBMGA005278*, which encodes an RR1-type cuticular protein, was expressed in the +/*bd* and *bd*/*bd* strains at 7.90- and 43.60-fold, respectively, of the level observed in the Dazao strain. Based on the RNA-seq data, the RPKM values were 20.26 and 117.15-fold, respectively, the value observed in the Dazao strain. This consistent pattern confirmed the validity of the expression data.

## Discussion

The ancestors of the cultivated silkworm species *B. mandarina* spent most of their lives as larvae with soft bodies. Accordingly, caterpillar has developed a wide range of mechanisms, such as coloration, to protect itself from predators^35^,^36^. The *bd* mutant silkworm has a melanized larval body color that is similar to that of *B. mandarina*.

We analyzed the differences between the transcriptome data obtained from the wild-type Dazao, *bd* and +/*bd* strains and screened for candidate genes potentially involved in *bd* larval melanism. At the same time, we sought to identify new genes involved in pigmentation. The transcriptome data obtained in this study revealed that the expressed genes represent approximately 51% of genes in the genome in the epidermis at the stop feeding stage (several hours prior to HCS), including ecdysone receptor (*ECR*) and ultraspiracle (*USP*)^37^, along with the pigment pattern regulation genes *optomotor-blind* (*omb*)^38^*, decapentaplegic* (*Dpp*)^39^, *apontic-like* (*apt-like*)^40^, and *abdominal-B* (*Abd-B*)^41^. In the DEG analysis, we screened 28 candidate DEGs, including 15 highly expressed cuticular proteins, in the *bd*/*bd* mutant. In addition, because *bd*/*bd* is an inbred line generated from the +/*bd* line, the 191 DEGs that were shared between *bd*/*bd* and +/*bd* were further analyzed. Of these, 58 DEGs were down-regulated and 133 up-regulated in *bd*/*bd* (Additional file 10: Fig. S5). Using manual annotation, we detected 23 cuticular proteins, 13 cecropin genes, 6 Osiris genes, and 25 enzymes, including laccase 2, a key melanism enzyme, and two major facilitator superfamily (MFS) proteins, that were up-regulated. The down-regulated genes included 22 enzymes, 2 MFS proteins, and 2 cuticular proteins.

Cuticular proteins are very important components of the epidermis, and more than 200 cuticular protein genes have been reported in *B. mori*^42^,^43^ The functions of most cuticular proteins have not yet been identified, although some have been associated with insect body shape, immunity and wing patterns^44^,^45^,^46^,^47^. Compared to the heterozygote, 23 cuticular proteins were found to be extremely significantly up-regulated in *bd*/*bd* individuals, including *BGIBMGA005277*, which was highly expressed (RPKM = 5963.64) during the early molting stage. The cuticular protein genes *BGIBMG000333, 002549, 011765, 014292, 002548, 008255, 000329, 011766* and *000341* (RPKMs >1000) were also expressed at very high levels in the *bd*/*bd* mutant. It was previously reported that the same cuticular protein genes have expression signals that vary in strength across different regions of the epidermis^48^, and many cuticular proteins are associated with marking specificity in butterflies^10^. The expression patterns of cuticular protein genes and colocalization between cuticular proteins and pigments suggest that pigmentation may be associated with cuticular protein expression. Although the mechanism by which cuticular proteins affect pigmentation is unclear, cuticular proteins are rendered stable and insoluble during cuticular sclerotization, and they resist enzyme degradation. When the pigment was incorporated into the cuticle, the cuticular proteins and pigments can form covalent crosslinks^49^. Based on data obtained from the *Bombyx mori* microarray database (www.silkdb.org/microarray/), the expression patterns of the 15 up-regulated cuticular proteins fluctuated, being highly expressed at the leaf-eating stage and expressed at lower levels during molting (Additional file 11, Fig. S6). However, these genes were expressed at very high levels during the early molting stage in the *bd* mutant. Therefore, we suggest that the 15 up-regulated cuticular proteins, which may interact with melanin and dopamine derivatives to form stable structures, may promote larval melanism. We hypothesize that the pigmentation of melanin granules requires the involvement of specific cuticular proteins and that greater numbers of melanin granules require more melanism-associated cuticular proteins. In addition, because of the limited scale of the larval epidermis, excessive accumulation of melanin, which requires more melanism-associated cuticular proteins, can cause physiological feedback, and the mechanism underlying this process may inhibit the expression of other cuticular protein genes.

We identified 13 antimicrobial peptide genes that were also expressed at higher levels in *bd*/*bd* individuals than in +/*bd* individuals. Antimicrobial peptides are important effectors of innate immunity in insects. Melanization is an important factor that is expressed by insect immune cells in the hemolymph, and when a pathogen invades an insect’s blood, it activates a serine protease cascade, resulting in the production of melanin. The deposition of phenol oxidase (*PO*) on exogenous microbial surfaces causes a vesicle to form, which can stop the growth and movement of exogenous microbes and isolate them from host tissues^50^. As previously reported for the mapping region of *Bm* (*Black moth*) and *Sw* (*Wild wing spot*), an antimicrobial peptide gene (*BGIBMGA005658*) has been suggested to be responsible for silkworm melanism mutants and the region associated with wing color patterns and body color in other lepidopteran species, including *Biston betularia* and *Heliconius butterflies*^49^. However, the mechanism by which antimicrobial peptides contribute to epidermal melanism is unclear.

Six Osiris genes were markedly up-regulated in the *bd*/*bd* mutant. The *Triplo-lethal Locus* (*Tpl*) causes the death in haploid and triploid cells in *Drosophila*^51^, with death mainly occurring during the embryonic stage. Osiris family members, the functions of which are unknown, all contain the DUF167 domain, which is present at this locus^52^, and the Osiris genes of *B. mori* are highly conserved with those in *Drosophila*. Nonetheless, the phenotypic contribution of the Osiris genes requires further analysis.

In our analysis, 25 genes encoding enzymes were up-regulated in the *bd*/*bd* mutant, including *laccase 2*, which is a key factor in melanin production. Pigment metabolism pathway enzymes, such as *TH*, *yellow, yellow-f,yellow-f2*, *ebony*, *DDC* and *PO* (*phenol oxidase*), were not detected because they are not yet expressed during the early stage of molting; however, *laccase 2* is expressed beginning on the third day of the fourth instar, which occurs earlier than molting^21^. Most of these enzymes are related to metabolic processes in DEGs. Although the activities of enzymes are clearly very important, achieving robust conclusions will require further study.

We identified two cuticular protein genes, *BGIBMGA012601* and *BGIBMGA000326* that were down-regulated, suggesting that these proteins may be negatively correlated with larval melanism, but this finding requires further research. A major facilitator superfamily (MFS) protein has been confirmed to transport small molecules in silkworms, and two loss-of-function mutations in the MFS protein are responsible for the coloration mutants *cheek and tail spot* (*cts*)^53^,^54^ and *red egg* (*re*)^55^, suggesting that this MFS protein participates in pigmentation. Two of the down-regulated genes encode aminopeptidase N-12, a type of metal protease, and aminopeptidase N. These proteins are mainly distributed in the kidney, the small intestine and respiratory tract epithelial cells in humans, in which they promote angiogenesis and relay signals^56^. However, their roles in the *bd*/*bd* mutant remain unknown. Although the associations between coloration and certain DEGs are unclear, they are apparently involved in development, biological metabolism and other processes. These DEGs may be related to *bd*/*bd* mutant female sterility or other phenotypes because it has been reported that insect melanism can affect fecundity by affecting energy distribution^57^. However, there were no significant differences between pupal and adult body colors between the *bd* mutant and wild-type silkworms. Thus, it is more likely that the effect on fertility reflects abnormal development (unpublished data).

In summary, we identified over 10,000 early-expressed genes and transcripts expressed in the fourth molting period in silkworms, a time point that represents an early stage of molting in which pigment synthesis enzyme genes are not yet being expressed. These data therefore provide a reference for screening upstream regulatory genes for their effect on color patterns. Additionally, using comparative analyses, we detected 28 genes that were potential candidate DEGs that may be involved in color patterns. These data may be useful in resolving the molecular mechanisms underlying the *bd* phenotype. These genes include 15 DEGs that encode cuticular proteins and that are very highly expressed in the *bd*/*bd* mutant. These cuticular protein genes may be required for melanism, acting as structural support and/or matter transporters to help pigment granules during pigmentation. Different pigment particles may require corresponding cuticular proteins to produce pigmentation. If cuticular protein expression levels are insufficient, the result is a correspondingly insufficient amount of pigment to fill the cuticle. We therefore propose that the cuticular proteins that are up-regulated in the *bd*/*bd* mutant may promote larval melanism in silkworms. The interactions between cuticular proteins and pigment particles will be assessed in future studies.

## Materials and Methods

### Silkworm strains and tissue collection

The *black dilute* (*bd*) mutant silkworm strains (homozygotes and heterozygotes) and the wild-type strain Dazao were obtained from the Silkworm Gene Bank of Southwest University, Chongqing, China. All larvae were reared on fresh mulberry leaves at 25 °C in 75% relative humidity under stable conditions (12 hours light:12 hours dark). Because *bd*/*bd* females are sterile, *bd* offspring were generated by crossing +/*bd* females with *bd*/*bd* males (+/*bd* ♀ × *bd*/*bd* ♂). The molting period was based on the feeding activity and HCS timing. The integuments were collected from 4 individuals from each strain on ice during the stage when feeding stops in the fourth molt. Other tissues, including the fat body, trachea, head, and appendages, were removed using 0.7% normal saline (NS) solution. Each mixture containing four integuments was washed quickly with 0.7% NS, blotted on filter paper, and then stored at −80 °C until RNA extraction was performed.

### RNA preparation and Illumina RNA-seq

Total RNA was prepared from a mixture of the entire integument of four individuals from each strain using TRIzol® reagent (Invitrogen, Carlsbad, CA, USA) according to the manufacturer’s protocol. RNA degradation and contamination were determined using 1% agarose gels. RNA purity was checked using a Nano-Photometer spectrophotometer (IMPLEN, CA, USA), and RNA integrity was assessed using an RNA Nano 6000 Assay Kit that was provided with a Bio-analyzer 2100 system (Agilent Technologies, CA, USA). A total of 3 μg of RNA per sample was used as the input material for RNA sample preparations. Sequencing libraries were generated using NEB Next Ultra™ RNA Library Prep Kit from Illumina (NEB, USA) according to the manufacturer’s instructions, and index codes were added to assign sequences to each sample. The clustering of the index-coded samples was performed using a cBot Cluster Generation System with TruSeq PE Cluster Kit v3-cBot-HS (Illumina) according to the manufacturer’s instructions. After cluster generation, the library preparations were sequenced using an Illumina HiSeq 2000 platform, and 100-bp paired-end reads were generated.

### Transcriptome data analysis

#### Quality control

The original image data obtained from high throughput sequencing were translated into sequencing reads using a base-calling program. The sequencing reads were Raw Data (raw reads), which were first processed in FASTQ format using in-house Perl scripts. In this step, Clean Data (clean reads) were obtained from the raw data by removing the reads containing adapters, reads containing poly-N, and low-quality reads. At the same time, the Q20, Q30 and GC contents of the clean data were calculated. All downstream analyses were based on high-quality clean data. The reference genome and the gene model annotation files were downloaded from the Silkworm Genome Database (SilkDB; http://www.silkdb.org/silkdb/). An index for the reference genome was generated using Bowtie v2.0.6^58^, and paired-end clean reads were aligned to the reference genome using TopHat v2.0.9^59^. We selected TopHat as the mapping tool because this program can generate a database of splice junctions based on the gene model annotation file. Thus, it produces better mapping results than other non-splice mapping tools.

#### Quantification of gene expression levels and differential expression analysis

HTSeq v0.6.1 was used to count the number of reads that mapped to each gene, and the RPKM of each gene was then calculated based on the length of the gene and the read counts that were mapped to that gene. RPKM simultaneously considers the effect of the sequencing depth and the gene length for the read counts and is currently the most commonly used method for estimating gene expression levels. Prior to the differential gene expression analysis, the read counts for each sequenced library were adjusted using the edgeR package and a scaling normalizing factor. Differential expression analyses were performed using two samples in the DEGSeq R package (1.12.0), and P-values were adjusted using the Benjamini and Hochberg method^59^. A corrected P-value of 0.005 and a log_2_ (fold-change) of 1 were set as the thresholds for significant differential expression.

#### GO and KEGG enrichment analyses of DEGs

The GO analysis of novel transcripts and highly expressed genes was plotted using the WEGO (Web Gene Ontology Annotation Plot) program (http://wego.genomics.org.cn/cgi-bin/wego/index.pl)^60^. The GO enrichment analysis of DEGs was implemented using the GOseq R package, in which gene length bias was corrected. GO terms with corrected P-values less than 0.05 were considered significantly enriched. KEGG is a database resource that is used to determine the high-level functions and utilities of biological systems, such as the cell, organism and ecosystem, from molecular-level information, especially large-scale molecular datasets that are generated using genome sequencing and other high-throughput experimental technologies (http://www.genome.jp/kegg/). We used KOBAS software to assess the statistically significant enrichment of DEGs in KEGG pathways.

#### Quantitative reverse transcription-PCR

The primers that were used for qRT-PCR were designed based on the consensus sequences of each alignment. qRT-PCR was performed using a CFX96™ Real-Time PCR Detection System (Bio-Rad, Hercules, CA) with SYBR Green qRT-PCR Mix (Bio-Rad). The cycling parameters were as follows: 95 °C for 3 min followed by 40 cycles of 95 °C for 10 s and annealing for 30 s (the primers are listed in Additional file 12: Table S6). Three biological repeats were performed for each genotype, and each sample was analyzed in triplicate. The gene expression levels were normalized against the expression levels of the ribosomal protein L3 (RpL3). The relative expression levels were analyzed using the classical R = 2^−ΔΔCt^ method^61^.

#### Availability of supporting data

The raw transcriptome reads obtained during this study have been deposited at NCBI Short Read Archive (SRA, http://www.ncbi.nlm.nih.gov/sra/) under the bioproject number PRJNA299880 and accession numbers SRR2839844, SRR2850623 and SRR2866061.

#### Ethics

In this research, only invertebrates (silkworms and wild silkworms) were used, and the research did not involve ethical and animal welfare issues.

## Additional Information

**How to cite this article**: Wu, S.Y. *et al.* Comparative analysis of the integument transcriptomes of the *black dilute* mutant and the wild-type silkworm *Bombyx mori*. *Sci. Rep.* **6**, 26114; doi: 10.1038/srep26114 (2016).

## Supplementary Material

Supplementary Dataset 1

Supplementary Dataset 2

Supplementary Dataset 3

Supplementary Dataset 4

Supplementary Dataset 5

## Figures and Tables

**Figure 1:**
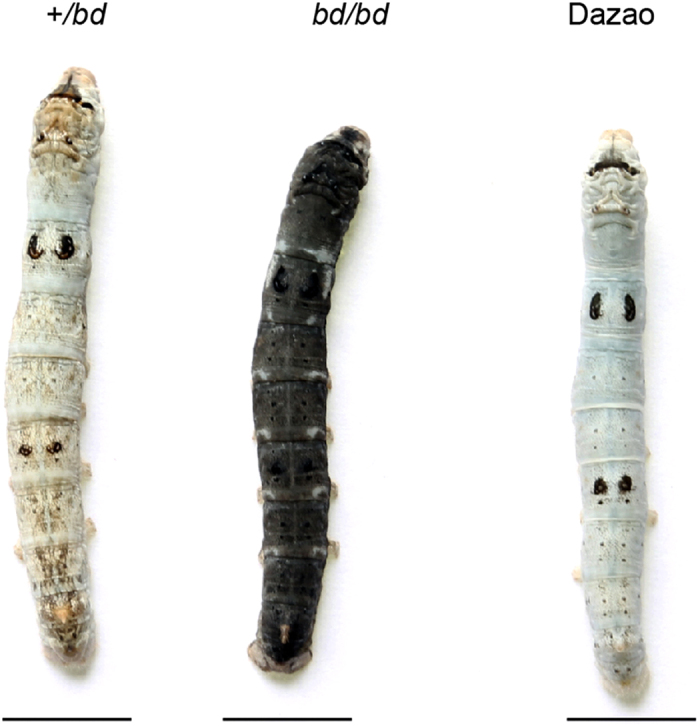
The phenotype of larvae at instar 5, day 3. The *black dilute (bd*/*bd)* larvae (middle) exhibited significantly more melanism than the wild-type Dazao (left) and the heterozygote (+/*bd*) (right). Scale bar, 1 cm.

**Table 1:** Summary of the sequence assembly after Illumina sequencing.

Sample name	Raw reads	Clean reads	Clean bases	Error rate (%)	Q20 (%)	Q30 (%)	GC content (%)
DZ_1	46054840	45494027	4.55G	0.03	96.46	92.52	49.45
DZ_2	46054840	45494027	4.55G	0.03	93.8	88.18	49.37
*bd*_1	46399908	45964807	4.6G	0.03	96.42	92.46	48.61
*bd*_2	46399908	45964807	4.6G	0.03	94.04	88.56	48.56
*bd*/+_1	53686247	53294746	5.33G	0.03	96.06	91.72	47.96
*bd*/+_2	53686247	53294746	5.33G	0.03	93.33	87.29	47.98

The numbers 1 and 2 at the end of the sample name represent the left and right ends (paired-end sequencing), respectively. Gb: gigabase. Q20: percentage of bases with a Phred value of at least 20. Q30: percentage of bases with a Phred value of at least 30.

**Table 2:** Distribution of transcript levels in the three silkworm strains.

RPKM Interval	Dazao	*bd*/*bd*	+/*bd*
0~1	11539 (49.56%)	11518 (49.47%)	11195 (48.08%)
1~3	1982 (8.51%)	2132 (9.16%)	2017 (8.66%)
3~15	5266 (22.62%)	5027 (21.59%)	4958 (21.29%)
15~60	3098 (13.30%)	3120 (13.40%)	3478 (14.94%)
>60	1400 (6.01%)	1488 (6.39%)	1637 (7.03%)

RPKM: Reads per kilobase per million reads. The ratios of the gene number to the total gene number are presented in parentheses.

**Table 3:** Functional annotation of DEGs.

Expression difference	Gene ID	Putative function	Microarray probe
	BGIBMGA000324*	cuticular protein RR-1 motif 46 precursor	sw08718
	BGIBMGA000328*	cuticular protein RR-1 motif 42 precursor	sw01201
	BGIBMGA000329*	putative cuticle protein	sw19893
	BGIBMGA000333*	larval cuticle protein LCP-22 precursor	sw01978
	BGIBMGA000341*	cuticular protein RR-1 motif 28 precursor	sw17751
	BGIBMGA002384*	cuticular protein glycine-rich 13 precursor	sw01437
	BGIBMGA002548*	cuticular protein RR-1 motif 4 precursor	sw06519
	BGIBMGA002549*	cuticular protein RR-1 motif 5 precursor	sw06436
	BGIBMGA004906	threo-3-hydroxyaspartate ammonia-lyase-like	
	BGIBMGA005277*	cuticular protein RR-1	sw00948
	BGIBMGA005278*	cuticular protein RR-1 motif 3 precursor	sw00947
	BGIBMGA005695	acid lipase-1	
Up-regulated	BGIBMGA007040	putative cysteine proteinase CG12163-like	
	BGIBMGA008255*	cuticular protein	sw22198
	BGIBMGA009184	putative peptidase	
	BGIBMGA011399	uncharacterized protein LOC101741978	
	BGIBMGA011765*	cuticular protein	sw22357
	BGIBMGA011766*	cuticular protein	sw18864
	BGIBMGA012968	Bm8-interacting protein 2d-4 precursor	
	BGIBMGA012997	glucose dehydrogenase [acceptor]-like	
	BGIBMGA013242	cis, cis-muconate transporter protein	
	BGIBMGA013656	solute carrier family 2, facilitated glucose transporter member 6-like	
	BGIBMGA013863	gloverin 1 precursor	
	BGIBMGA014292*	cuticular protein	sw18864
	Novel01073*	cuticular protein CPFL	
	Novel01661	nimrod B	
Down-regulated	BGIBMGA010763	aminopeptidase N-12	
	BGIBMGA010764	aminopeptidase N-12	

Overlapping DEGs were searched using the BLAST nr database and FlyBase, and putative functions were annotated to identify the best hits. An asterisk indicates a cuticular protein. Microarray probes were searched against the *Bombyx mori* microarray database (www.silkdb.org/microarray/).
