# Supplementary material for: Comparative analysis of the integument transcriptomes of the black dilute mutant and the wild-type silkworm Bombyx mori
Source: Sci Rep. 2016 May 19;6:26114. doi: 10.1038/srep26114 (PMC4872147; doi:10.1038/srep26114)
Supplement: Supplementary Dataset 1 [file srep26114-s1.doc]

**Supplementary information**

**Comparative analysis of the integument transcriptomes of the *black dilute* mutant and the wild-type silkworm *Bombyx mori***

**Songyuan Wu^1^, Xiaoling Tong^1^, Chenxing Peng^1^, Gao Xiong^1^, Kunpeng Lu^1^, Hai hu^1^, Duan Tan^1^, Chunlin Li^1^, Minjin Han^1^, Cheng Lu^1*^, Fangyin Dai^1*^**

^1^State Key Laboratory of Silkworm Genome Biology, Key Laboratory for Sericulture Functional Genomics and Biotechnology of Agricultural Ministry, Southwest University, Chongqing 400715, China

Songyuan Wu, E-mail: jademars@sina.com;

Xiaoling Tong, E-mail: [xltong@swu.edu.cn](mailto:xltong@swu.edu.cn);

Chenxing Peng, E-mail: [mxtaste@sina.cn](mailto:mxtaste@sina.cn);

Gao Xiong, E-mail: xgao13@163.com;

Kunpeng Lu, E-mail: lkp123@swu.edu.cn;

Hai Hu, E-mail: [huhaiswu@163.com](mailto:huhaiswu@163.com);

Duan Tan, E-mail: tanzeduan@163.com;

Chunlin Li, E-mail: lclin13@163.com;

Minjin Han, E-mail: minjinhan@126.com.

^*^Corresponding authors

E-mail: fydai@swu.edu.cn

or lucheng@swu.edu.cn

State Key Laboratory of Silkworm Genome Biology

Southwest University

Chongqing, China 400715

Tel.: 0086-023-68250793

Fax: 0086-023-68251128

**Additional file 1: Figure S1. The phenotype of *bd***/***bd* and wild silkworms at instar 5, day 3.**

**
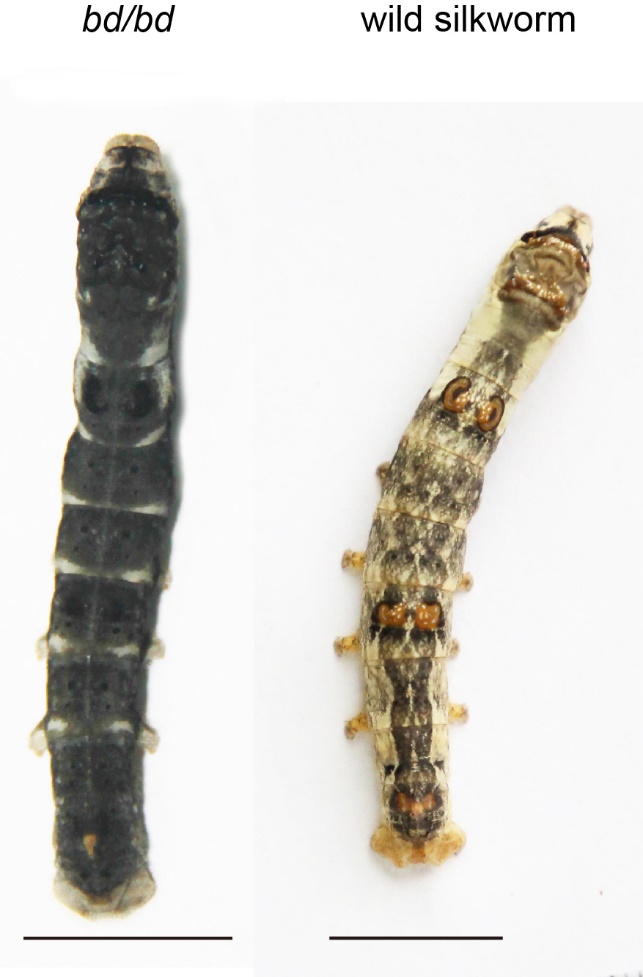
**

A wild silkworm captured from Ankang, Shaanxi Province, is shown on the right, and a *black dilute (bd*/*bd)* larva is shown on the left. Scale bar, 1 cm.

**Additional file 2: Figure S2. Melanin metabolism in silkworm.**


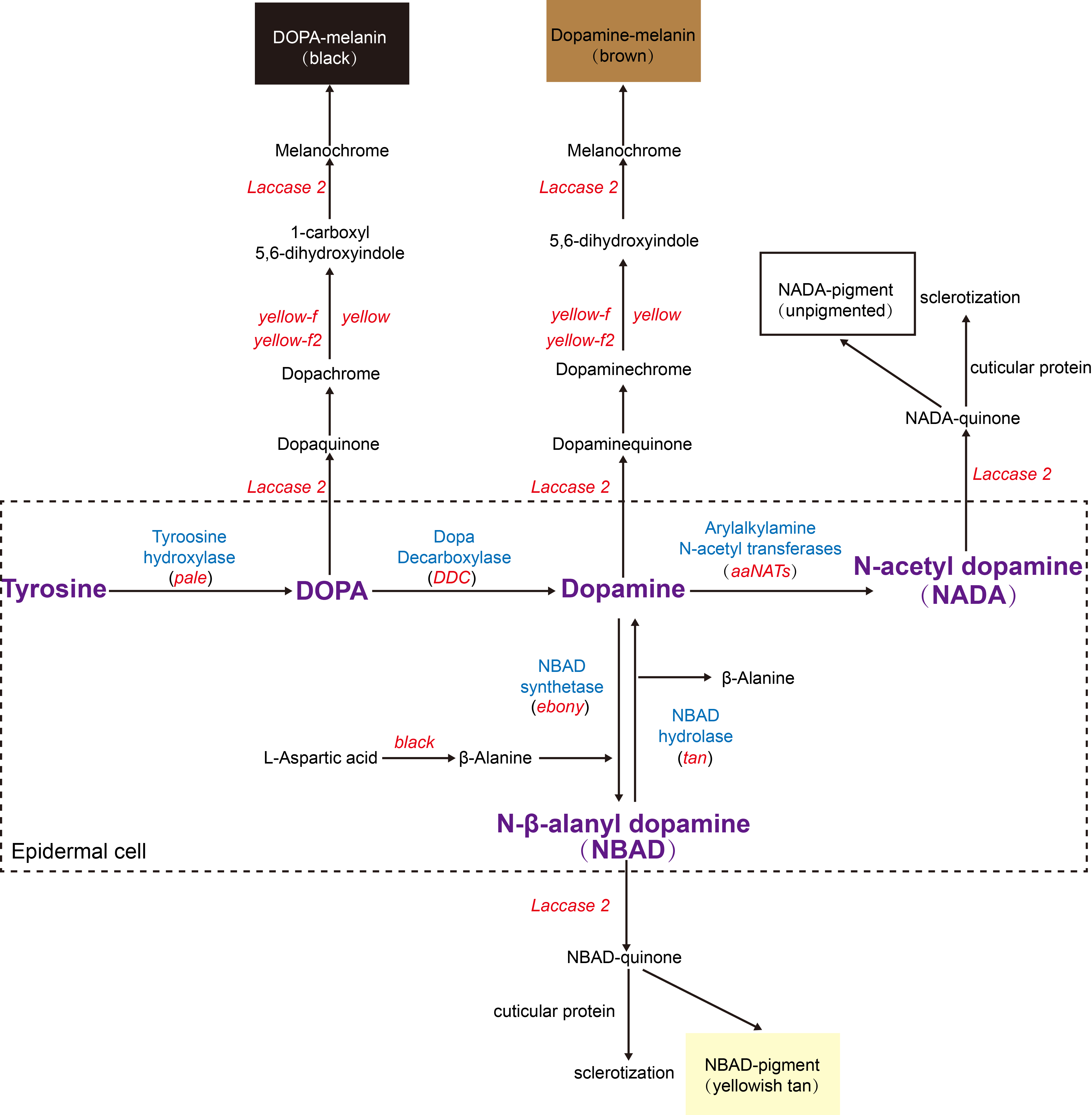


Pigment precursors are indicate in purple, enzymes are shown in blue, and the genes that encode them are shown in red.

**Additional file 3: Table S1. Reference sequence mapping.**

| Sample name | Dazao | *bd*/*bd* | *+*/*bd* |
| --- | --- | --- | --- |
| Total reads | 90988054 | 91929614 | 1.07E+08 |
| Total mapped | 83483951 (91.75%) | 74683487 (81.24%) | 84421894 (79.2%) |
| Multiple mapped | 1942754 (2.14%) | 1891283 (2.06%) | 2206229 (2.07%) |
| Uniquely mapped | 81541197 (89.62%) | 72792204 (79.18%) | 82215665 (77.13%) |
| Read-1 | 41357057 (45.45%) | 36945940 (40.19%) | 41914513 (39.32%) |
| Read-2 | 40184140 (44.16%) | 35846264 (38.99%) | 40301152 (37.81%) |
| Reads map to '+' | 40792536 (44.83%) | 36363579 (39.56%) | 41133206 (38.59%) |
| Reads map to '-' | 40748661 (44.78%) | 36428625 (39.63%) | 41082459 (38.54%) |
| Non-splice reads | 58795560 (64.62%) | 51824402 (56.37%) | 58441561 (54.83%) |
| Splice reads | 22745637 (25%) | 20967802 (22.81%) | 23774104 (22.3%) |

We mapped clean reads to the *B. mori* reference genome, Silkworm Genome Database ([www.silkdb.org/silkdb/](http://www.silkdb.org/silkdb/)). The proportion of total reads in the three silkworm transcriptome libraries that mapped to the genome ranged from 79.20% to 91.75%。

**Additional file 6: Figure S3. GO analysis of novel genes.**

**
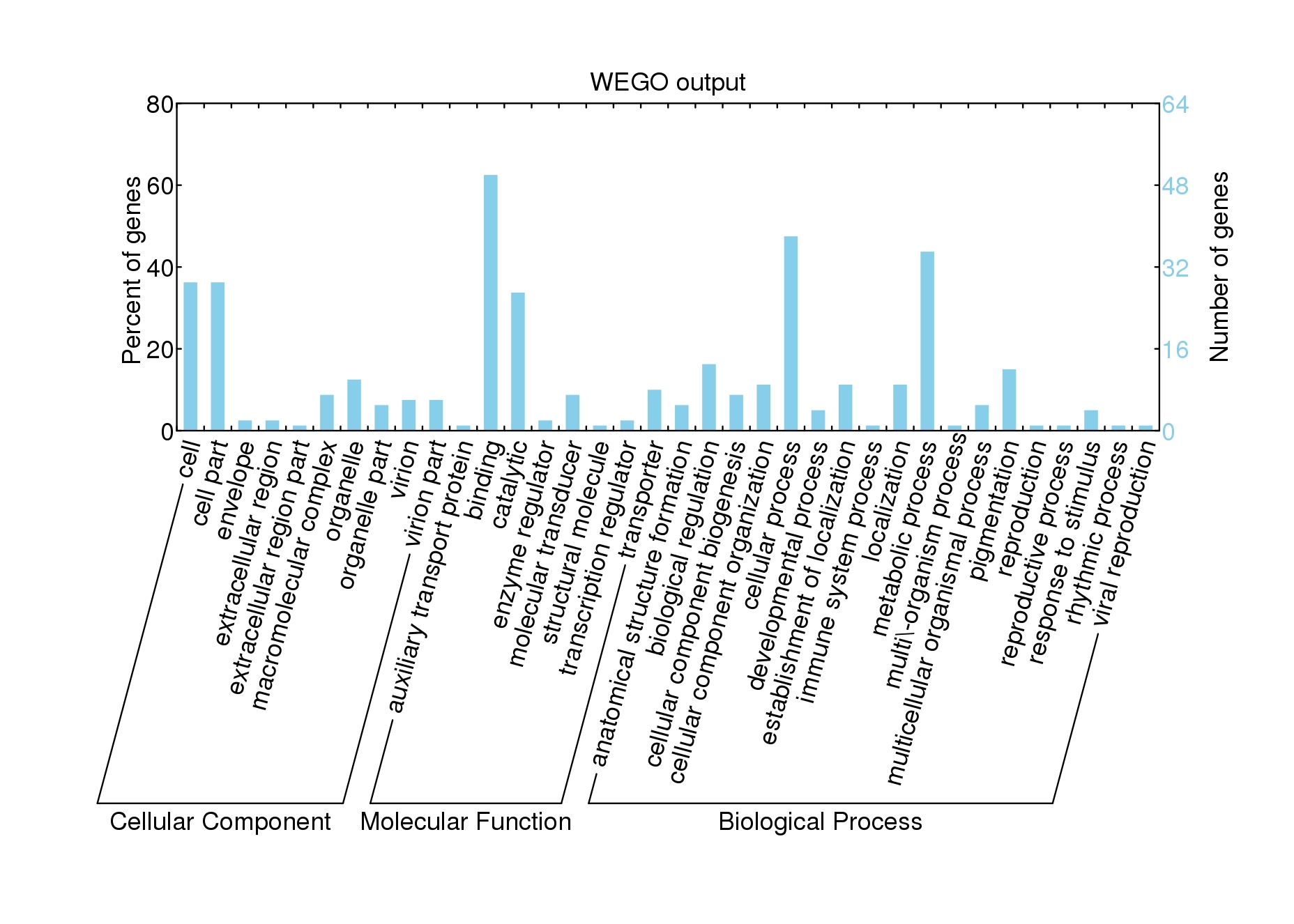
**

The x-axis shows the 2^nd^-level GO term, and the y-axis shows the percentage and number of genes; 80 genes with GO terms were annotated.


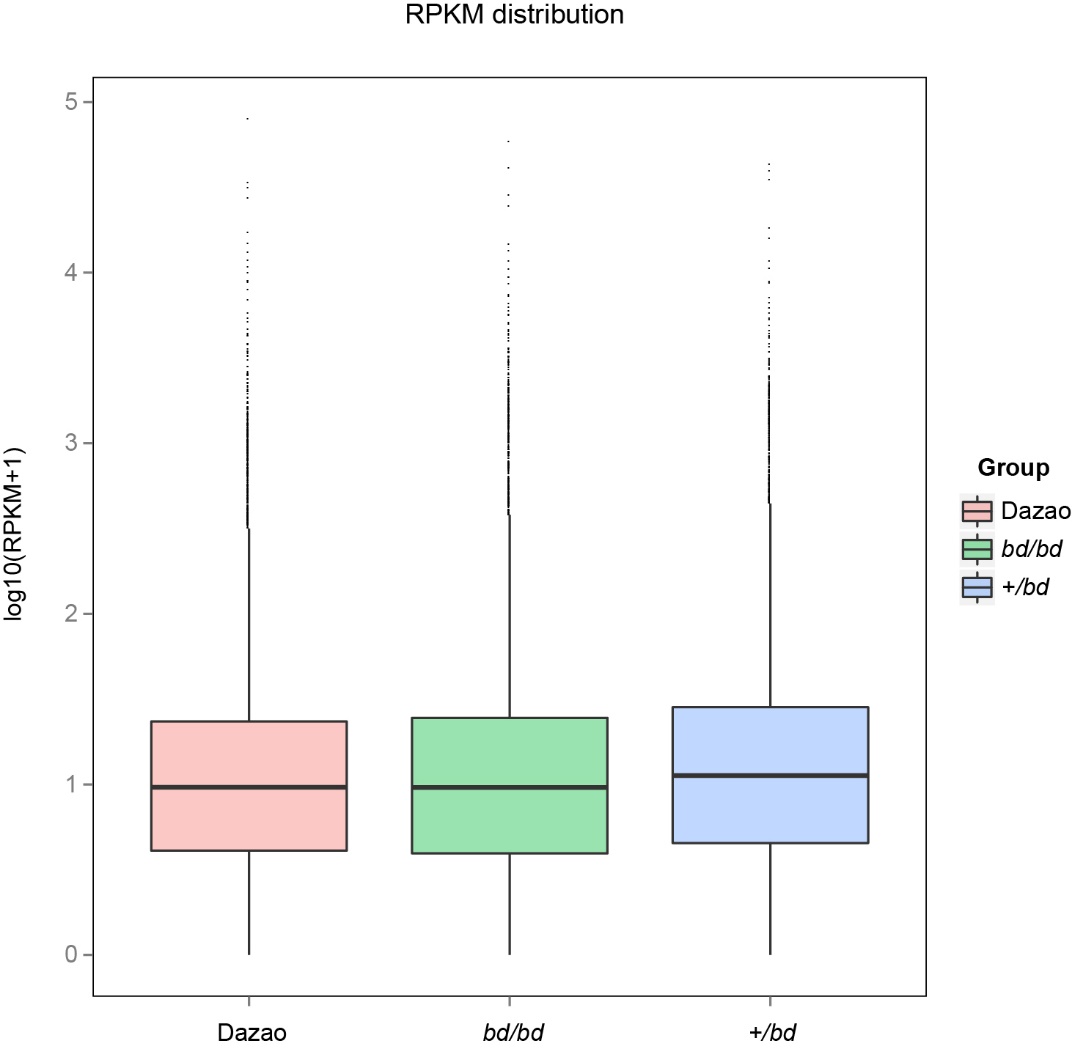
**Additional file 7: Figure S4. Boxplot of log-transformed RPKM expression values across the three silkworm strains.**

RPKM: Reads per kilobase per million reads. The solid horizontal line represents the median, and the box extends from the lower quartile to the upper quartile.

**Additional file 10: Figure S5. Volcano plot of DEGs between *bd***/***bd* and *+***/***bd.***

*
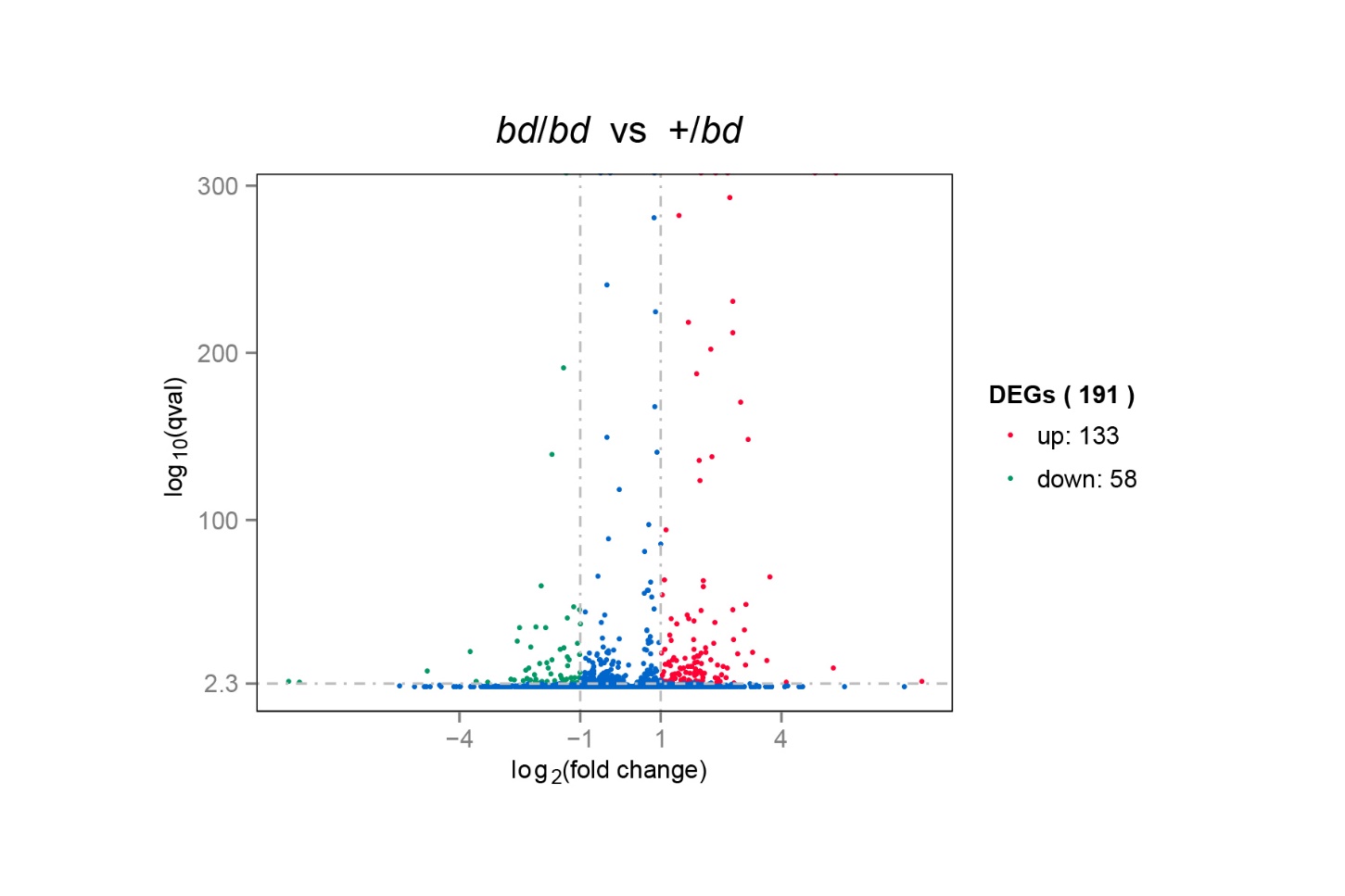
*

Red points indicate up-regulation; green points indicate down-regulation.

**Additional file 11: Figure S6. Expression patterns of the cuticular protein genes in different larval stages based on microarray data**
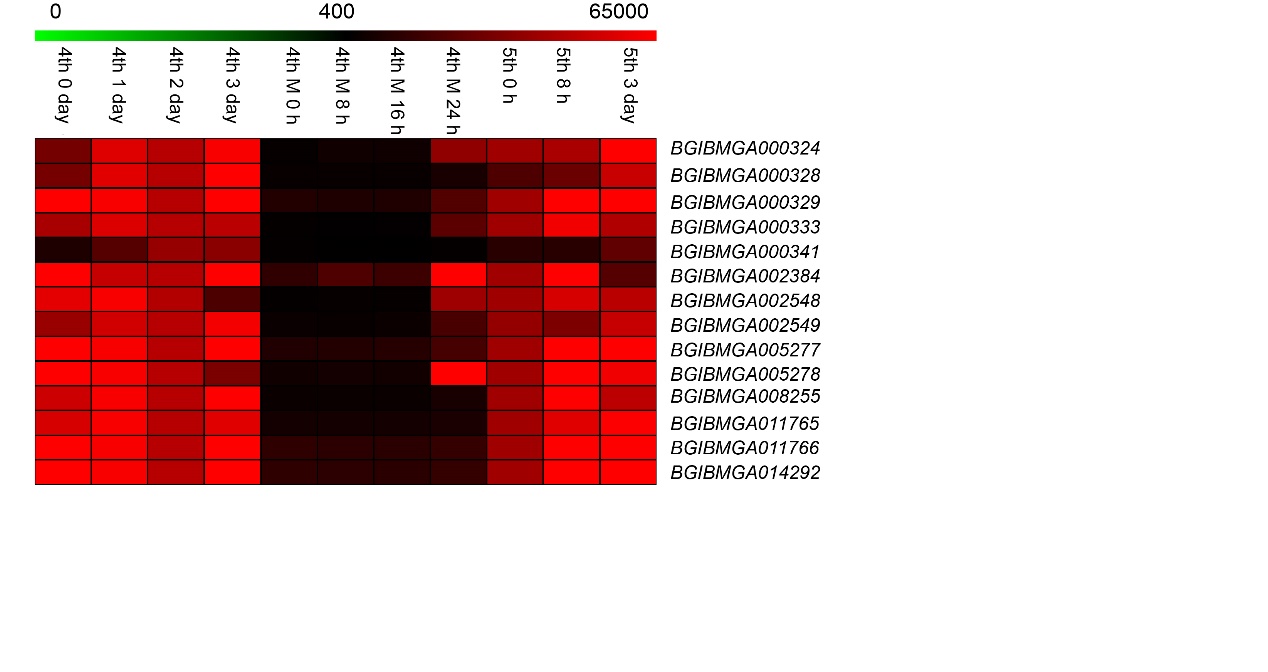
**.**

Of the 28 DEGs, 14 cuticular proteins could be tracked using microarray data (Table 3). 4^th^: fourth instar, 5^th^: fifth instar, M: molting, h: hours.

**Additional file 12: Table S6. List of qRT-PCR primers.**

| Accession number/  gene name | Forward primer (5'-3') | Reverse primer (5'-3') |
| --- | --- | --- |
| *BGIBMGA001325* | AAGCCCTTGGACCCGTTACA | GGAAGCACCAACAGTTTACCG |
| *BGIBMGA000010* | AGACTCTGACCCGAACATCCTG | GGGTGAGCCTTTGCTGTCC |
| *BGIBMGA000013* | TTACCCGACGAGCCCAGAG | CGGCGGCAAAGGCAAT |
| *BGIBMGA000023* | TTCGTCTTCGCTCTGGTGC | GCTTTAGCCGAACCGAGGA |
| *BGIBMGA003510* | GCTTCTTCCGCCGACACCT | GCTCGCATGTTGCTTCCTTT |
| *BGIBMGA003874* | CCACCCGCATAGCACAAACA | AAGAACGACGGACGATTTGC |
| *BGIBMGA005277* | CCGCCCCAGTGGTCAAAT | GTCGGCAGATGGCTTCCCT |
| *BGIBMGA005278* | AGAACTTACATCTTCCTCGCTTTG | CACTGATTCCGTTGCTGGTC |
| *BGIBMGA006288* | CCTGTTCCACCTCCGCATAA | TTCTCGCTGGCTGATGTTTGA |
| *BGIBMGA006874* | CACGGCAGACGGGAGGAA | GCGTCCATAAAGCGGTACACCTA |
| *BGIBMGA009743* | CGCACTTCGGAGACTGGC | CCGGAATCTTTGGCTCCTT |
| *BGIBMGA010240* | AACGCCCTCACGACCAATG | CCACCAGCCTCTTTGTTGATG |
| *BGIBMGA011765* | TCAGAGTCCTTGTCGCATTCG | GCGGTGTAGTCGTAGGTGGAAG |
| *BGIBMGA011766* | GATTCTTGTTACCCTCGCCACT | CAACGGAGGTGGTGAGAGC |
| *BGIBMGA005320* | ACATTTCGGCGGAGGGAT | GGACCTCCATCTATGCGTTGT |
| RpL3 | CGGTGTTGTTGGATACATTGAG | GCTCATCCTGCCATTTCTTACT |

The GenBank accession number for RpL3 is NM_001043661. The accession numbers beginning with BGI are from the Silkworm Genome Database (http://www.silkdb.org/silkdb/).
